# Changes in salivary biomarkers of oxidative status in calves at weaning and grouping

**DOI:** 10.1186/s12917-021-03087-2

**Published:** 2021-12-04

**Authors:** Camila Peres Rubio, Damián Escribano, Eva Mainau, José Joaquin Cerón, Elena Navarro, Xavier Manteca

**Affiliations:** 1 Department of Animal and Food Science, School of Veterinary Science, Universitat Autònoma de Barcelona, 08193 Cerdanyola del Vallès, Barcelona, Spain; 2 Interdisciplinary Laboratory of Clinical Analysis (Interlab-UMU), Veterinary School, Campus of Excellence Mare Nostrum, University of Murcia, Campus de Espinardo s/n, 30100 Murcia, Spain

**Keywords:** Antioxidants, Cupric, Ferric, Oxidant, Stress

## Abstract

**Background:**

Saliva is being increasingly used as a sample for measuring biomarkers in several species and shows a high potential of use to detect and monitor stress. The weaning and grouping in dairy calves are a particularly stressful time. Therefore, the objectives of this study were to evaluate a panel of antioxidant and oxidant biomarkers in the saliva of calves on the day of weaning (W0), 2 days after weaning or milk withdrawal (W + 2), and 4 days after grouping (G + 4). In addition, to verify if cortisol and oxytocin concentrations are related to the biomarkers measured.

**Results:**

Salivary cupric reducing antioxidant capacity (CUPRAC), ferric reducing ability of saliva (FRAS), Trolox equivalent antioxidant capacity (TEAC), advanced oxidation protein products (AOPP), and ferrous oxidation-xylenol orange (FOX) were significantly higher (*P* < 0.02) 4 days after grouping than the day of weaning and 2 days after. The increases were 50 and 54% for CUPRAC, 93 and 116% for FRAS, 117 and 135% for TEAC, 22 and 49% for AOPP and 10 and 5% for FOX in comparison with weaning and 2 days after, respectively. In addition, oxytocin and cortisol showed significant negative and positive correlations (*P* < 0.05) respectively with the biomarkers of oxidative status.

**Conclusions:**

Our results showed that calves after grouping show increases in antioxidants and oxidants concentrations, indicating that a balance between these molecules has been tried to maintain during this stressful situation. The dynamic changes of biomarkers of oxidative status should be explored and characterised in other stressful conditions.

**Supplementary Information:**

The online version contains supplementary material available at 10.1186/s12917-021-03087-2.

## Background

Biomarkers of oxidative status include those analytes that can measure or estimate antioxidant or oxidant compounds. These biomarkers can detect situations in which the antioxidant system cannot counteract the overproduction of oxidants, such as reactive oxygen species (ROS) [1]. These situations can lead to oxidative damage of molecules such as lipids, proteins, DNA and RNA, and are related to various diseases in humans and animals [2–4].

Traditionally oxidative stress biomarkers in ruminants have been measured in blood, and their variations have been associated with diseases. For example, changes in these biomarkers have been described in cows with metabolic disorders such as ketosis [5], or reproductive disorders like ovarian follicular cyst and endometritis [6, 7], in bulls with severe symptoms of foot-and-mouth disease [8], and in sheep during parasitism infection [9, 10]. In addition, different situations that can produce stress in ruminants have also been associated with changes in antioxidant and oxidant biomarkers, a condition also called “oxidative eustress” [11]. For example, in lactating dairy cows during thermal stress [12] or the transition from late lactation and into the early dry period [13] and shearing in sheep [14]. The mechanism involved remains unclear, but it seems the changes in oxidative biomarkers could be, in part, mediated by stress-related hormones such as cortisol [15, 16].

Saliva is being increasingly used as a sample to measure biomarkers in several species [17–19]. This is due to various facts, such as its non-invasive and inexpensive collection and composition, that can reflect dynamic changes in the body [20]. In particular, biomarkers of oxidative status such as total antioxidant capacity (Trolox equivalent antioxidant capacity [TEAC], ferric reducing ability of saliva [FRAS], cupric reducing antioxidant capacity [CUPRAC]), uric acid, thiol, advanced oxidation protein products (AOPP) and hydrogen peroxide (H_2_O_2_) have been measured in the saliva of cows [21, 22].

The weaning in dairy calves is a period of diet change and a new physical and social environment [23] which can result in adverse effects on both production and wellbeing [24], being a particularly stressful time [25]. In the same way, the grouping of calves can lead to aggression and social stress [26, 27].

In this study, we hypothesised that the biomarkers of oxidative stress could change in the saliva of calves during stress situations such as weaning and grouping, and that these changes could be related to those produced in biomarkers of welfare and stress such as oxytocin and cortisol. Therefore, the objectives of this work were to evaluate a panel of antioxidant (CUPRAC, FRAS, TEAC, and uric acid) and oxidant biomarkers (AOPP, ferrous oxidation-xylenol orange [FOX] and peroxide-activity [POX-Act]) in the saliva of calves on the day of the weaning (W0), 2 days after weaning (W + 2) or milk withdrawal and 4 days after grouping (G + 4). In addition, the correlation of these biomarkers with stress biomarkers such as cortisol and oxytocin were evaluated.

## Results

### Biomarkers of oxidative status

**Fig. 1:**
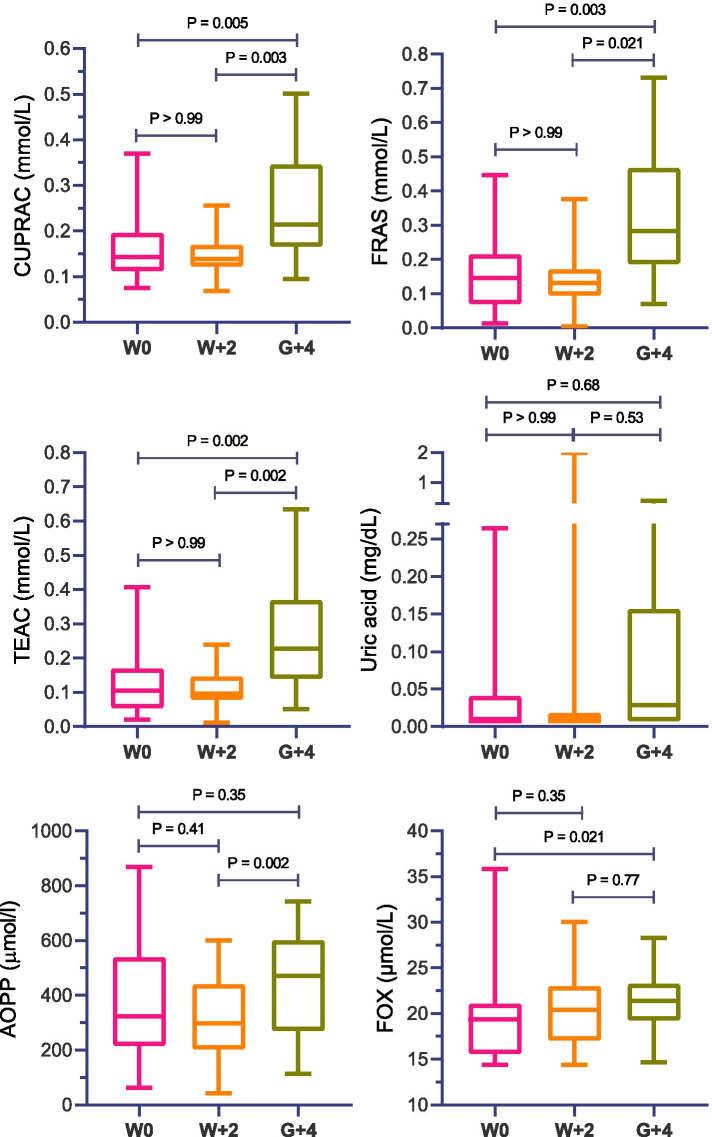
Salivary cupric reducing antioxidant capacity (CUPRAC), ferric reducing ability of saliva (FRAS), Trolox equivalent antioxidant capacity (TEAC), uric acid, advanced oxidation protein products (AOPP), and ferrous oxidation-xylenol orange (FOX) of 25 calves on the day of weaning (W0), 2 days after weaning or milk withdrawal (W + 2) and 4 days after grouping calves (G + 4)

The results obtained in the measured biomarkers of oxidative status in the calves throughout the study are presented in Fig. 1. On the antioxidant side, a significant increase (*P* < 0.021) was observed 4 days after grouping calves (G + 4) when compared to the day of weaning (W0) and 2 days after weaning or milk withdrawal (W + 2) in the concentrations of CUPRAC (which showed a 50 and 54% increase compared with W0 and W + 2, respectively), FRAS (93 and 116% increase compared with W0 and W + 2, respectively), and TEAC (117 and 135% increase compared with W0 and W + 2, respectively). Uric acid did not show differences between the time-points of the study (*P* > 0.05). Regarding the oxidants, the POX-Act results were below the detection limit in saliva and data is not shown. Salivary AOPP concentrations were significantly higher at G + 4 than W + 2 (49% increase; *P* = 0.002). FOX showed higher concentrations at G + 4 when compared with W0 (10% increase; *P* = 0.021).

### Cortisol

**Fig. 2:**
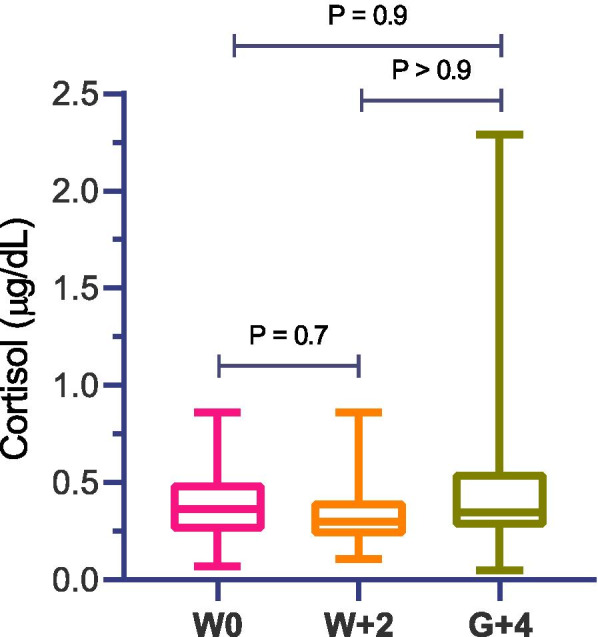
Cortisol in saliva of calves on the day of the weaning (W0), 2 days after weaning or milk withdrawal (W + 2) and 4 days after grouping calves (G + 4)

The results of salivary cortisol are shown in Fig. 2. No significant changes were observed in the cortisol concentrations during all the periods of study (*P* < 0.05).

### Correlation study

The correlations between the different biomarkers studied, the salivary cortisol and the previously reported values of salivary oxytocin [16] are shown in Table 1. Significant and negative correlations with oxytocin were found with CUPRAC (*r* = − 0.43, *P* < 0.0001), FRAS (*r* = − 0.38, *P* < 0.0006), TEAC (*r* = − 0.40, *P* < 0.0003) and AOPP (*r* = − 0.30, *P* = 0.007). Regarding cortisol, positive and significant correlations were observed with CUPRAC (*r* = 0.25, *P* = 0.028), FRAS (*r* = 0.29, *P* = 0.011), TEAC (*r* = 0.31, *P* = 0.005) and AOPP (*r* = 0.40, *P* = 0.0003).

**Table 1:** Correlation coefficients between oxidative status biomarkers and oxytocin and cortisol concentrations in the calves on the day of the weaning, after weaning and after grouping (*n* = 25)

Biomarker of oxidative status	Oxytocin	Cortisol
CUPRAC	−0.435***	0.252*
FRAS	−0.385***	0.290*
TEAC	−0.402***	0.314**
Uric acid	−0.123	−0.043
AOPP	−0.307**	0.409***
FOX	−0.189	0.014

*CUPRAC* cupric reducing antioxidant capacity, *FRAS* ferric reducing ability of saliva, *TEAC* Trolox equivalent antioxidant capacity, *AOPP* advanced oxidation protein products, *FOX* ferrous oxidation-xylenol orange

* *P* < 0.05; ** *P* < 0.01; *** *P* < 0.001

## Discussion

This study is the first that determines a panel of biomarkers of oxidative status in the saliva of calves during productive and stressful situations such as weaning and grouping. Also, the possible relationship between those biomarkers and biomarkers of stress and welfare such as cortisol and oxytocin in the saliva of calves are evaluated.

The increase in the oxidant biomarkers observed in our work in the saliva of the calves 4 days after grouping would reflect an oxidative challenge. Possible links between stressful situations and the increase in oxidant compounds have been reported. It seems that excessive production of oxygen radicals through the higher formation of xanthine oxidase occurs during stressful stimuli [28]. In addition, any stressful situations can activate the hypothalamic-pituitary-adrenal axis followed by releasing the corresponding stress hormones such as cortisol and corticosterone [29], and increase the presence of ROS in cell cultures [30], which could be one of the mechanisms over stress led to the overproduction of oxidants as observed in this study in saliva. In this line, increases in oxidants after stressful conditions in animals have been described, such as increased serum and plasma lipid peroxides in horses and calves after transportation [31, 32].

The increased antioxidant biomarkers found in our study would aim to counteract the oxidants’ increase after the stressful condition. This has been described in previous reports in which stressful situations have been related to lipid peroxidation and consequently increased antioxidant activity as an adaptation to the stimulus [33, 34]. In agreement with our results, salivary oxidant and antioxidant biomarkers were increased in sheep after an acute stress stimulus such as shearing [19]. In addition, increases in antioxidants in blood in stressful situations have been described in different species. For example, in dogs, the transportation triggered the activation of antioxidant response in serum [35]. In cows, increased oxidants and mobilisation of blood antioxidants have been reported after weaning and heat stress [36, 37]. The occurrence of a state of oxidative stress in calves may increase the potential and incidence of infectious and metabolic diseases, which denotes that the measurement of biomarkers of oxidative status would be of help to improve the management practices for these animals [36].

Oxytocin and cortisol hormones have been associated with positive and negative welfare, respectively, in calves and other species [16, 38–40]. In our study, although no significant change has been observed in the salivary cortisol, salivary oxytocin correlated negatively with the oxidant and antioxidant biomarkers, whereas cortisol showed a positive correlation. Although the correlations were moderate, they were all highly significant statistically. The positive correlation between the biomarkers of oxidative status and cortisol can be explained by the activation of the hypothalamic-pituitary-adrenal axis as described above, while the negative correlation with oxytocin could be due to its decrease in situations of stress or negative emotions [16].

This study has limitations that deserve consideration. The biomarkers results have not been corrected by salivary protein, and additional studies should be performed in the future about the possible need for correction by protein in biomarkers of oxidative status [41, 42]. Only females have been included in the experimental design, thence further investigation about the sex-specific differences in oxidative stress biomarkers in saliva should be done. Furthermore, the effects of the dietary changes during the experiment have not been considered, and ideally, a control group without grouping after weaning should have been included. Besides, studies including sampling times before than 24 h after weaning would allow evaluation of the oxidative response in an acute situation of weaning. In the same way, sampling in a longer time than 4 days of grouping would help to find when the concentrations of the biomarkers achieve the concentration observed at weaning.

## Conclusions

In this report, we demonstrated a significant increase in oxidant biomarkers such as AOPP and FOX and antioxidant biomarkers such as CUPRAC, FRAS, and TEAC in the saliva of calves after a stressful situation like grouping. Based on these preliminary results, it could be concluded that stress situations would promote an increase in oxidant molecules, which is compensated by an increase in antioxidants, a situation that could be assessed by measuring biomarkers of oxidative status in the saliva. The dynamic changes of biomarkers of oxidative status should be explored and characterised in other stressful conditions.

## Material and methods

### Animals, housing, and general management

The trial was in conformity with the ARRIVE guidelines [43]. Twenty-five Friesian female calves from multiparous and primiparous dams from a commercial farm were studied, split into 6 replicates (or weeks). Each replicate included from 2 to 7 calves. Calves were separated from dams as soon as possible after calving. The mean ± ES interval time between calving and calf-dam separation was 28.86 ± 4.64 min. Calves were moved from the maternity pen to individual hutches (1.2 m × 1.8 m) that were bedded with straw within a 24-hutches, 6-row outdoor calf facility. As soon as calves were allocated in the hutches, they received 4 L of colostrum by oesophageal feeder tube. Then, calves fed pasteurized milk from the tank of the farm at 8:00 am and 4:00 pm by individual calf buckets. The following quantities were given to each calf: 6 L/d from day 2 to day 14, 8 L/d from day 15 to day 42, 6 L/d from day 43 to day 49 and 4 L/d from day 50 until weaning (gradual reduction of milk supply). In compliance with management practices on the farm, two criteria had to be met to wean calves: (1) calves with at least 8 weeks of life (from day 57 to day 63 after birth) and (2) calves with body weight (BW) ≥ 80 Kg. Every Tuesday at 12:00 pm, calves that were expected to be weaned (at least 8 weeks of life), were individually weighted. For calves meeting the criteria (BW ≥ 80 Kg), milk was withdrawn in the afternoon. Calves with BW < 80 Kg, were fed milk (4 L/day) until reached 80 Kg for milk withdrawal. In the present work, 19 calves were weaned at 8 weeks of life and 6 calves needed one additional week for reaching at least 80 Kg (9 weeks of life). Mean ± ES of weight at weaning (milk withdrawal) was 87.01 ± 0.78 Kg and calves had 60.96 ± 0.86 days of life. After 3 days of milk withdrawal (Friday), calves were moved to a group pen of 8–10 calves each. Calves were transported within the same farm a distance of 1200 m by a small truck.

Freshwater was offered to calves starting at 1 d of age. Water was changed twice daily after milk administration. Calves had free access to a starter diet 1 week after birth. Calves were offered with a 300 g of starter diet 1 week after birth. Every day at 12:00 pm, the starter was checked and changed ensuring that calves were fed ad libitum. The starter diet was formulated based on NRC recommendations [44]. After grouping, calves were fed TMR mixed with the same starter diet for 15 days (See Additional file).

#### Saliva collection

**Fig. 3:**
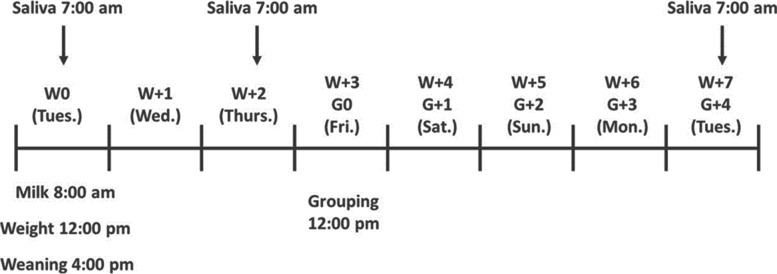
Timeline of saliva sampling according to weaning (milk withdrawal; W) and grouping calves to pens (G)

Saliva samples were obtained from all animals at three time-points: on the day of the weaning (W0), 2 days after weaning or milk withdrawal (W + 2) and 7 days after weaning that corresponded to 4 days after grouping calves (G + 4). All samples were obtained between 7:00 and 8:00 am (before morning milk feeding or TMR mixed administration) (Fig. 3). Saliva collections were performed using a cotton swab attached through a Kocher clip, and calves were allowed to chew it for 1 min. Then, the cotton swab was placed into Salivette tubes (Sarstedt, Aktienge-sellschaft & Co. Nümbrecht, Germany) and centrifuged at 6000 rpm for 12 min. Approximately 1 to 2 mL of saliva from each cotton swab were stored in Eppendorf tubes and frozen at − 80 °C until analysis.

### Oxidative stress biomarkers

About antioxidant biomarkers determination, CUPRAC was measured as previously described [45] and is based on the capacity of the sample in reducing cupric to cuprous ion. The FRAS was performed following the method described by Benzie and Strain [46], which measures the ferric to ferrous ion reduction by the sample. The TEAC, based on the inhibition of the radical ABTS by the sample, was performed according to the assay described by Arnao et al. [47]. Finally, uric acid was measured using a commercially available spectrophotometric method (Uric Acid reagent OSR6698 Beckman Coulter AU analysers, Switzerland).

Regarding oxidant biomarkers evaluation, AOPP was based on the determination of oxidatively modified albumin and di-tyrosine containing cross-linked proteins as previously described [48]. The FOX was assessed based on the automatic determination of the ferrous oxidation by xylenol orange as described by Arab and Steghens [49]. Finally, the POX-Act was performed based on the oxidation of the chromogen substrate 3,5,3′5’-tetramethylbenzidine (TMB) by the reaction of horseradish peroxidase (HRP) with the sample as previously described [50].

All analyses were done at Murcia University using an automated biochemistry analyser (Olympus AU400 Automatic Chemistry Analyzer, Olympus Europe GmbH, Germany). All assays showed inter-and intra-assay imprecision of < 15% and showed high linearity (*r*^2^ > 0.95) after serial sample dilutions.

### Cortisol assay

The cortisol concentration in saliva was determined by the use of a solid-phase, competitive chemiluminescent enzyme immunoassay that used a polyclonal rabbit anti-cortisol antibody (Immulite/Immulite 1000 cortisol; Siemens Medical Solutions Diagnostics, Deerfield, IL, USA).

### Statistical analysis

Statistical analyses were performed using Graph Pad Software Inc. (GraphPad Prism, version 6 for Windows, Graph Pad Software Inc., San Diego, USA). Statistical significance was set at *P* < 0.05. The normality of data was verified using the Shapiro-Wilk test. All biomarkers’ results, except AOPP, were not normally distributed. Analysis of variance (ANOVA) of repeated measures with Dunn’s multiple comparisons test was used to compare the non-parametric results of the sequential time samplings during the study. The ANOVA followed by Tukey’s multiple comparison test was applied to study the possible presence of differences between AOPP results. Spearman’s correlation coefficient (r) was calculated between the oxidative stress biomarkers with cortisol and the oxytocin values obtained from these animals in a previous report [16] (W0, median: 780 pg/mL; W + 2, median: 795 pg/mL; G + 4, median: 18.6 pg/mL). An r value of < 0.3 was considered a negligible correlation, following the rule of thumb [51]. Results were described as median and 25th–75th percentile (in the text) and line-box plots (in Figures).

## Supplementary Information

**Additional file 1.** Description of the chemical composition of the dry matter (DM) basis of the starter offered to calves in the experiment.

## Data Availability

The datasets used and/or analysed during the current study are available from the corresponding author on reasonable request.

## Abbreviations

ABTS: 2,2′-azino-bis(3-ethylbenz-thiazoline-6-sulfonic acid
ANOVA: Analysis of variance
AOPP: Advanced oxidation protein products
CUPRAC: Cupric reducing antioxidant capacity
FOX: Ferrous oxidation-xylenol orange
FRAS: Ferric reducing ability of saliva
H_2_O_2_: Hydrogen peroxide
HRP: Horseradish peroxidase
POX-Act: Peroxide-activity
ROS: Reactive oxygen species
TEAC: Trolox equivalent antioxidant capacity
TMB: 3,5,3′5’-tetramethylbenzidine.
