# Supplementary material for: Changes in salivary biomarkers of oxidative status in calves at weaning and grouping
Source: BMC Vet Res. 2021 Dec 4;17:373. doi: 10.1186/s12917-021-03087-2 (PMC8642975; doi:10.1186/s12917-021-03087-2)
Supplement: Supplementary file 1 — Additional file 1. Description of the chemical composition of the dry matter (DM) basis of the starter offered to calves in the experiment. [file 12917_2021_3087_MOESM1_ESM.docx]

**Additional file:** Description of the chemical composition of the dry matter (DM) basis of the starter offered to calves in the experiment.

| Chemical composition | DM |
| --- | --- |
| UFL | 1,088 IU/kg |
| NED | 1,871 Mcal/Kg |
| Protein | 23,123 % |
| TDN | 182,669 g/Kg |
| RUP | 8,457 % |
| Fat | 3,011 % |
| Ash | 7,497 % |
| Crude fibre | 7,266 % |
| ADF | 9,409 % |
| NDF | 18,472 % |
| NFC | 47,897 % |
| Calcium | 1,104 % |
| Phosphorus | 0,495 % |
| Magnesium | 0,236 % |
| Potassium | 1,148 % |
